# Supplementary material for: Staphylococcus aureus Breast Implant Infection Isolates Display Recalcitrance To Antibiotic Pocket Irrigants
Source: Microbiol Spectr. 2022 Dec 12;11(1):e02884-22. doi: 10.1128/spectrum.02884-22 (PMC9927092; doi:10.1128/spectrum.02884-22)

Supplemental Material

Table S1. MBCs of antibiotics tested against *S. aureus* and *P. aeruginosa* strains

	MBC(ug/mL)			
	Gentamicin	Cefazolin	Bacitracin	TAPI
JE2	1	6	>32	Susceptible
117	1	0.5	>32	Susceptible
158	1	1	>32	Susceptible
PA01	6	>32	>1400	Susceptible
157	4	>32	>1400	Susceptible
160	6	>32	>1400	Susceptible

Table S2. Antimicrobial Resistance Genes Annotated in BIAI Isolates

Strain	ST Type	Genes	Resistance Category
JE2	8	mecA	penam
		mecR1	penam
		tet(38)	tetracycline
		mepR	glycylcycline;tetracycline
		mepA	glycylcycline;tetracycline
		mgrA	acridine_dye;cephalosporin;fluoroquinolone;penam;peptide;tetracycline
		Staphylococcus_aureus_norA	fluoroquinolone
		arlS	acridine_dye;fluoroquinolone
		arlR	acridine_dye;fluoroquinolone
		Staphylococcys_aureus_LmrS	aminoglycoside;diaminopyrimidine;macrolide;oxazolidinone;phenicol
		Staphylococcus_aureus_FosB	fosfomycin
117	39	arlR	acridine_dye; fluoroquinolone
		arlS	acridine_dye; fluoroquinolone
		mgrA	acridine_dye; cephalosporin;

			fluoroquinolone; penam; peptide; tetracycline
		mepA	glycylcycline; tetracycline
		mepR	glycylcycline; tetracycline
		tet(38)	tetracycline
		FosB	fosfomycin
158	45	mgrA	acridine_dye; cephalosporin; fluoroquinolone; penam; peptide; tetracycline
		arlS	acridine_dye; fluoroquinolone
		arlR	acridine_dye; fluoroquinolone
		mepA	glycylcycline; tetracycline
		mepR	glycylcycline; tetracycline
		tet(38)	Tetracycline
		PC1_beta-lactamase_(blaZ)	Penam
		msrA	lincosamide; macrolide; oxazolidinone; phenicol; pleuromutilin; streptogramin; tetracycline
		mphC	macrolide
PAO1	549	TriA	triclosan
		TriB	triclosan
		TriC	triclosan
		MexA	aminocoumarin;carbapenem;cephalosporin;cephamycin;diaminopyrimidine;fluoroquinolone;macrolide;monobactam;penam;penem;peptide;phenicol;sulfonamide;tetracycline
		MexB	aminocoumarin;carbapenem;cephalosporin;cephamycin;diaminopyrimidine;fluoroquinolone;macrolide;monobactam;penam;penem;peptide;phenicol;sulfonamide;tetracycline
		OprM	acridine_dye;aminocoumarin;aminoglycoside;carbapenem;cephalosporin;cephamycin;diaminopyrimidine;fluoroquinolone;macrolide;monobactam;penam;penem;peptide;phenicol;sulfonamide;tetracycline
		Pseudomonas_aeruginosa_catB7	phenicol
		fosA	fosfomycin
		PmpM	aminoglycoside;benzalkonium_chloride;fluoroquinolone
		mexM	phenicol
		mexN	phenicol
		mexY	acridine_dye;aminoglycoside;carbapenem;cephalosporin;cephamycin;fluoroquinolone;macrolide;penam;phenicol;tetracycline
		mexX	acridine_dye;aminoglycoside;carbapenem;cephalosporin;cephamycin;fluoroquinolone;macrolide;penam;phenicol;tetracycline
		Pseudomonas_aeruginosa_soxR	acridine_dye;cephalosporin;fluoroquinolone;glycylcycline;penam;phenicol;rifamycin;tetracycline;triclosan
		MexE	diaminopyrimidine;fluoroquinolone;phenicol

		MexF	diaminopyrimidine;fluoroquinolone;phenicol
		OprN	diaminopyrimidine;fluoroquinolone;phenicol
		OpmB	aminocoumarin;macrolide;monobactam;tetracycline
		MuxC	aminocoumarin;macrolide;monobactam;tetracycline
		MuxB	aminocoumarin;macrolide;monobactam;tetracycline
		MuxA	aminocoumarin;macrolide;monobactam;tetracycline
		Pseudomonas_aeruginosa_CpxR	aminocoumarin;aminoglycoside;carbapenem;cephalosporin;cephamycin;diaminopyrimidine;fluoroquinolone;macrolide;monobactam;penam;penem;peptide;phenicol;sulfonamide;tetracycline
		opmE	acridine_dye;carbapenem;diaminopyrimidine;macrolide;phenicol;tetracycline
		mexQ	acridine_dye;carbapenem;diaminopyrimidine;macrolide;phenicol;tetracycline
		mexP	acridine_dye;carbapenem;diaminopyrimidine;macrolide;phenicol;tetracycline
		arnA	peptide
		mexK	macrolide;tetracycline;triclosan
		mexJ	macrolide;tetracycline;triclosan
		mexL	macrolide;tetracycline;triclosan
		ArmR	aminocoumarin;carbapenem;cephalosporin;cephamycin;diaminopyrimidine;fluoroquinolone;macrolide;monobactam;penam;penem;peptide;phenicol;sulfonamide;tetracycline
		PDC-1	carbapenem;cephalosporin;monobactam
		APH(3')-IIb	aminoglycoside
		bcr-1	bicyclomycin
		mexG	acridine_dye;fluoroquinolone;tetracycline
		mexH	acridine_dye;fluoroquinolone;tetracycline
		mexI	acridine_dye;fluoroquinolone;tetracycline
		opmD	acridine_dye;fluoroquinolone;tetracycline
		mexV	acridine_dye;fluoroquinolone;macrolide;phenicol;tetracycline
		mexW	acridine_dye;fluoroquinolone;macrolide;phenicol;tetracycline
		OprJ	aminocoumarin;aminoglycoside;cephalosporin;diaminopyrimidine;fluoroquinolone;macrolide;penam;phenicol;tetracycline
		MexD	aminocoumarin;aminoglycoside;cephalosporin;diaminopyrimidine;fluoroquinolone;macrolide;penam;phenicol;tetracycline
		MexC	aminocoumarin;aminoglycoside;cephalosporin;diaminopyrimidine;fluoroquinolone;macrolide;penam;phenicol;tetracycline
		basS	peptide

		OpmH	triclosan
		Pseudomonas_aeruginosa_emrE	aminoglycoside
		OXA-50	cephalosporin;penam
157	633	MexC	aminocoumarin; aminoglycoside; cephalosporin; diaminopyrimidine; fluoroquinolone; macrolide; penam; phenicol; tetracycline
		MexD	aminocoumarin; aminoglycoside; cephalosporin; diaminopyrimidine; fluoroquinolone; macrolide; penam; phenicol; tetracycline
		OprJ	aminocoumarin; aminoglycoside; cephalosporin; diaminopyrimidine; fluoroquinolone; macrolide; penam; phenicol; tetracycline
		CrpP	fluoroquinolone
		mexW	acridine_dye;fluoroquinolone;macrolide;phenicol;tetracycline
		mexV	acridine_dye;fluoroquinolone;macrolide;phenicol;tetracycline
		arnA	peptide
		mexP	acridine_dye;carbapenem;diaminopyrimidine;macrolide;phenicol;tetracycline
		mexQ	acridine_dye;carbapenem;diaminopyrimidine;macrolide;phenicol;tetracycline
		opmE	acridine_dye;carbapenem;diaminopyrimidine;macrolide;phenicol;tetracycline
		Pseudomonas_aeruginosa_CpxR	aminocoumarin;aminoglycoside;carbapenem;cephalosporin;cephamycin;diaminopyrimidine;fluoroquinolone;macrolide;monobactam;penam;penem;peptide;phenicol;sulfonamide;tetracycline
		MuxA	aminocoumarin;macrolide;monobactam;tetracycline
		MuxB	aminocoumarin;macrolide;monobactam;tetracycline
		MuxC	aminocoumarin;macrolide;monobactam;tetracycline
		OpmB	aminocoumarin;macrolide;monobactam;tetracycline
		OprN	diaminopyrimidine;fluoroquinolone;phenicol
		MexF	diaminopyrimidine;fluoroquinolone;phenicol
		MexE	diaminopyrimidine;fluoroquinolone;phenicol
		Pseudomonas_aeruginosa_soxR	acridine_dye;cephalosporin;fluoroquinolone;glycylcycline;penam;phenicol;rifamycin;tetracycline;triclosan
		mexX	acridine_dye;aminoglycoside;carbapenem;cephalosporin;cephamycin;fluoroquinolone;macrolide;penam;phenicol;tetracycline
		mexY	acridine_dye;aminoglycoside;carbapenem;cephalosporin;cephamycin;fluoroquinolone;macrolide;penam;phenicol;tetracycline
		mexN	phenicol
		mexM	phenicol

		PmpM	aminoglycoside;benzalkonium_chloride;fluoroquinolone
		fosA	fosfomycin
		Pseudomonas_aeruginosa_catB7	phenicol
		mexK	macrolide; tetracycline; triclosan
		mexJ	macrolide; tetracycline; triclosan
		mexL	macrolide; tetracycline; triclosan
		ArmR	aminocoumarin; carbapenem; cephalosporin; cephamycin; diaminopyrimidine; fluoroquinolone; macrolide; monobactam; penam;penem; peptide;phenicol; sulfonamide; tetracycline
		PDC-3	carbapenem; cephalosporin; monobactam
		APH(3')-IIb	aminoglycoside
		bcr-1	bicyclomycin
		mexG	acridine_dye; fluoroquinolone; tetracycline
		mexH	acridine_dye; fluoroquinolone; tetracycline
		mexI	acridine_dye; fluoroquinolone; tetracycline
		opmD	acridine_dye; fluoroquinolone; tetracycline
		OprM	acridine_dye; aminocoumarin; aminoglycoside; carbapenem; cephalosporin; cephamycin; diaminopyrimidine; fluoroquinolone; macrolide; monobactam; penam;penem; peptide; phenicol; sulfonamide; tetracycline
		MexB	aminocoumarin; carbapenem; cephalosporin; cephamycin; diaminopyrimidine; fluoroquinolone; macrolide; monobactam; penam;penem; peptide; phenicol; sulfonamide; tetracycline
		MexA	aminocoumarin; carbapenem; cephalosporin; cephamycin; diaminopyrimidine; fluoroquinolone; macrolide; monobactam; penam;penem; peptide; phenicol; sulfonamide; tetracycline
		TriC	triclosan
		TriB	triclosan
		TriA	triclosan
		OXA-486	cephalosporin;penam
		Pseudomonas_aeruginosa_emrE	aminoglycoside
		OpmH	triclosan
		basS	peptide
160	633	mexK	macrolide;tetracycline;triclosan
		mexJ	macrolide;tetracycline;triclosan
		mexL	macrolide;tetracycline;triclosan
		ArmR	aminocoumarin;carbapenem;cephalosporin;cephamycin; diaminopyrimidine;fluoroquinolone;macrolide;monobactam;penam;penem;peptide;phenicol;sulfonamide;tetracycline
		PDC-3	carbapenem;cephalosporin;monobactam

		APH(3')-IIb	aminoglycoside
		bcr-1	bicyclomycin
		mexG	acridine_dye;fluoroquinolone;tetracycline
		mexH	acridine_dye;fluoroquinolone;tetracycline
		mexI	acridine_dye;fluoroquinolone;tetracycline
		opmD	acridine_dye;fluoroquinolone;tetracycline
		OprM	acridine_dye;aminocoumarin;aminoglycoside;carbapenem;cephalosporin;cephamycin;diaminopyrimidine;fluoroquinolone;macrolide;monobactam;penam;penem;peptide;phenicol;sulfonamide;tetracycline
		MexB	aminocoumarin;carbapenem;cephalosporin;cephamycin;diaminopyrimidine;fluoroquinolone;macrolide;monobactam;penam;penem;peptide;phenicol;sulfonamide;tetracycline
		MexA	aminocoumarin;carbapenem;cephalosporin;cephamycin;diaminopyrimidine;fluoroquinolone;macrolide;monobactam;penam;penem;peptide;phenicol;sulfonamide;tetracycline
		TriC	triclosan
		TriB	triclosan
		TriA	triclosan
		OXA-486	cephalosporin;penam
		Pseudomonas_aeruginosa_emrE	aminoglycoside
		OpmH	triclosan
		basS	peptide
		MexC	aminocoumarin;aminoglycoside;cephalosporin;diaminopyrimidine;fluoroquinolone;macrolide;penam;phenicol;tetracycline
		MexD	aminocoumarin;aminoglycoside;cephalosporin;diaminopyrimidine;fluoroquinolone;macrolide;penam;phenicol;tetracycline
		OprJ	aminocoumarin;aminoglycoside;cephalosporin;diaminopyrimidine;fluoroquinolone;macrolide;penam;phenicol;tetracycline
		CrpP	fluoroquinolone
		mexW	acridine_dye;fluoroquinolone;macrolide;phenicol;tetracycline
		mexV	acridine_dye;fluoroquinolone;macrolide;phenicol;tetracycline
		arnA	peptide
		mexP	acridine_dye;carbapenem;diaminopyrimidine;macrolide;phenicol;tetracycline
		mexQ	acridine_dye;carbapenem;diaminopyrimidine;macrolide;phenicol;tetracycline

		opmE	acridine_dye;carbapenem;diaminopyrimidine;macrolide;phenicol;tetracycline
		Pseudomonas_aeruginosa_CpxR	aminocoumarin;aminoglycoside;carbapenem;cephalosporin;cephamycin;diaminopyrimidine;fluoroquinolone;macrolide;monobactam;penam;penem;peptide;phenicol;sulfonamide;tetracycline
		MuxA	aminocoumarin;macrolide;monobactam;tetracycline
		MuxB	aminocoumarin;macrolide;monobactam;tetracycline
		MuxC	aminocoumarin;macrolide;monobactam;tetracycline
		OpmB	aminocoumarin;macrolide;monobactam;tetracycline
		OprN	diaminopyrimidine;fluoroquinolone;phenicol
		MexF	diaminopyrimidine;fluoroquinolone;phenicol
		MexE	diaminopyrimidine;fluoroquinolone;phenicol
		Pseudomonas_aeruginosa_soxR	acridine_dye;cephalosporin;fluoroquinolone;glycylcycline;penam;phenicol;rifamycin;tetracycline;triclosan
		mexX	acridine_dye;aminoglycoside;carbapenem;cephalosporin;cephamycin;fluoroquinolone;macrolide;penam;phenicol;tetracycline
		mexY	acridine_dye;aminoglycoside;carbapenem;cephalosporin;cephamycin;fluoroquinolone;macrolide;penam;phenicol;tetracycline
		mexN	phenicol
		mexM	phenicol
		PmpM	aminoglycoside;benzalkonium_chloride;fluoroquinolone
		fosA	fosfomycin
		Pseudomonas_aeruginosa_catB7	phenicol

Figure S1. Shared Virulence Factors Among the *Staphylococcus aureus* strains.

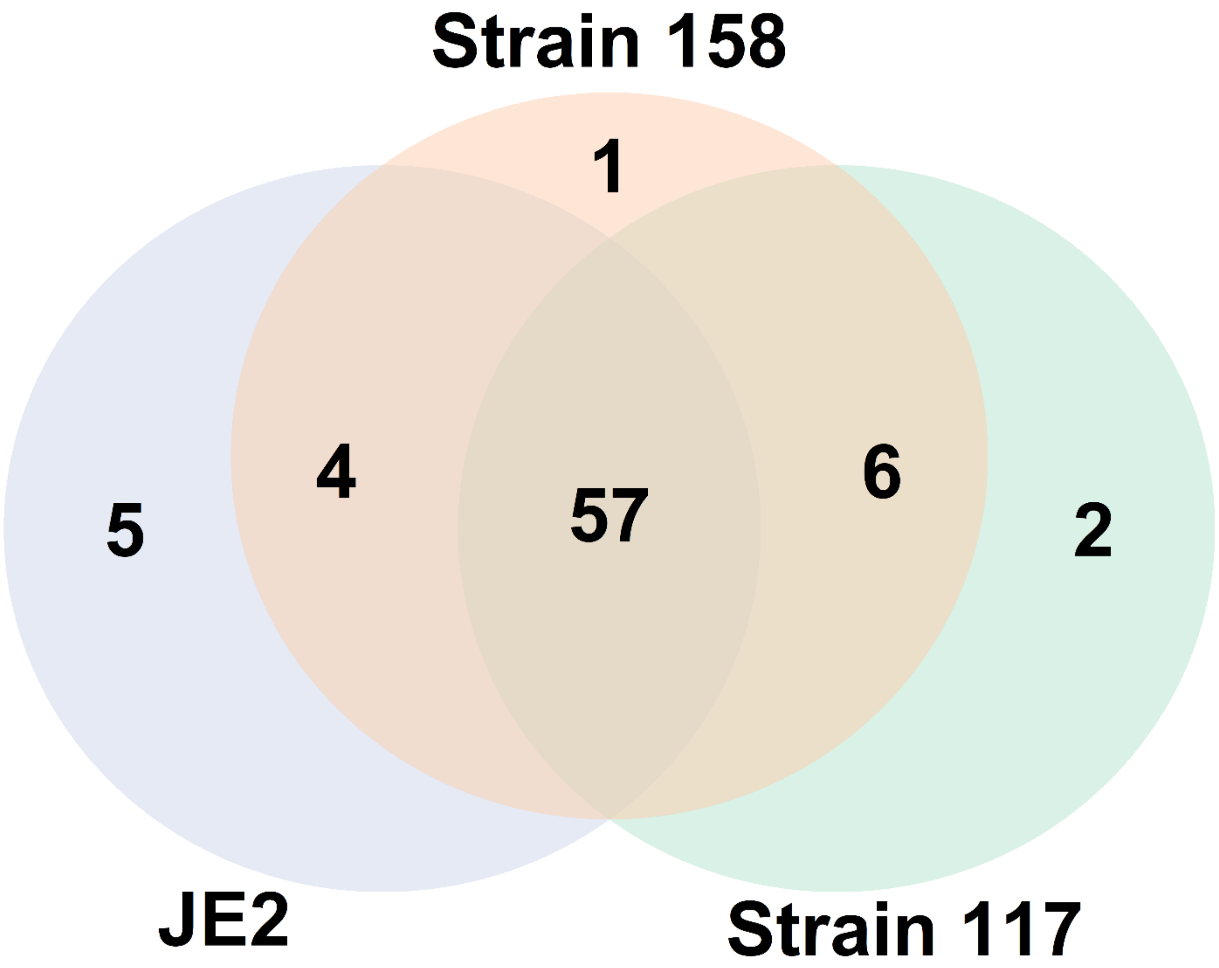

Supplement: Supplemental file 1 — Tables S1 and S2 and Fig. S1. Download spectrum.02884-22-s0001.pdf, PDF file, 0.2 MB [file spectrum.02884-22-s0001.pdf]
